# Dynamics of the Pre-Powerstroke Myosin Lever Arm and the Effects of Omecamtiv Mecarbil

**DOI:** 10.3390/ijms251910425

**Published:** 2024-09-27

**Authors:** Matthew Carter Childers, Michael Regnier

**Affiliations:** Department of Bioengineering, University of Washington, Seattle, WA 98195, USA; mcc7fb@uw.edu

**Keywords:** myosin, molecular dynamics, muscle structure and function, muscle regulation

## Abstract

The binding of small molecules to sarcomeric myosin can elicit powerful effects on the chemomechanical cycle, making them effective therapeutics in the clinic and research tools at the benchtop. However, these myotropes can have complex effects that act on different phases of the crossbridge cycle and which depend on structural, dynamic, and environmental variables. While small molecule binding sites have been identified crystallographically and their effects on contraction studied extensively, small molecule-induced dynamic changes that link structure–function are less studied. Here, we use molecular dynamics simulations to explore how omecamtiv mecarbil (OM), a cardiac myosin-specific myotrope, alters the coordinated dynamics of the lever arm and the motor domain in the pre-powerstroke state. We show that the lever arm adopts a range of orientations and find that different lever arm orientations are accompanied by changes in the hydrogen bonding patterns near the converter. We find that the binding of OM to myosin reduces the conformational heterogeneity of the lever arm orientation and also adjusts the average lever arm orientation. Finally, we map out the distinct conformations and ligand–protein interactions adopted by OM. These results uncover some structural factors that govern the motor domain–tail orientations and the mechanisms by which OM primes the pre-powerstroke myosin heads.

## 1. Introduction

Class II myosins are the motor proteins responsible for contractile force generation in striated muscle via cyclic ATP-dependent interactions with actin [1,2,3]. The contractile kinetics of muscle tissues are primarily determined by the chemomechanical properties of the myosin isoforms within sarcomeres [4] but can be fine-tuned by regulatory proteins and other factors, including small molecules. Myosin-targeted small molecules (i.e., myotropes) are being developed to address cardiomyopathies by directly targeting actin–myosin crossbridge cycling kinetics. Such myotropes include omecamtiv mecarbil (OM) [5], mavacamten (Mava) [6], aficamten (Afi) [7], and danicamtiv (Dani) [8]. Mava is approved for the treatment of obstructive hypertrophic cardiomyopathy (HCM) [9,10]; Afi significantly improved end point measures for patients vs placebo in the SEQUOIA-HCM trial [11] and is currently under investigation in other clinical trials (MAPLE-HCM and ACACIA-HCM); Dani has demonstrated promising effects as a myosin activator [8,12,13] and is being studied in further clinical trials; and OM was investigated as a therapy for patients with symptomatic heart failure in the GALACTIC-HF clinical trial and showed a modest improvement in the primary outcomes for patients, but did not significantly improve the overall patient survival or quality of life [14,15]. Independent of clinical efficacy, OM, Mava, Dani, and Afi all modulate crossbridge cycling kinetics: a greater understanding of the impact of these small molecules on myosin structure and dynamics could pave the way for the development of more efficacious and targeted compounds.

OM has calcium-dependent, competing activating and inhibitory effects on the crossbridge cycle, as discussed in detail by Kampourakis et al. [16]; briefly, OM enhances calcium sensitivity but decreases the cooperativity of activation [16,17,18]. The ultimate effects of OM modulate the number of myosin heads that form weakly-bound crossbridges but depend on regulatory structural changes in the thick and thin filaments. The OM–myosin interaction has been previously characterized in three X-ray crystallography studies. Winkelmann et al. [19] crystallized a chimeric construct containing the human β-cardiac motor domain (residues 1–787) fused to GFP in a nucleotide-free, extended near-rigor state of myosin. The authors conclude that the OM binds to a pocket at the junction between the converter domain/lever arm and the N-terminal domain that is natively present in the apo state. The Houdusse group also crystallized OM free/bound bovine β-cardiac myosin + essential light chain constructs in 2017 [20] and 2023 [21], both in the pre-powerstroke state. In these structures, the OM binds in the same region as in the Winkelmann et al. study [19], but due to the differences in lever arm positioning, the binding pocket geometry and the OM–myosin interactions are conformation-dependent, implying that the OM’s conformation adapts to variable lever arm orientations. All the structural studies indicate that OM stabilizes a primed lever arm orientation of myosin. 

Computational studies have previously investigated the effects of OM on myosin dynamics. Hashem et al. performed conventional molecular dynamics (MD) simulations of the Winkelmann et al. (PDB: 4P7H and 4PA0) apo (OM-free) and holo (OM-bound) OM [22] and found that the binding of OM to near-rigor myosin dampened the conformational fluctuations at the converter domain/lever arm hinge, increased the dynamic correlations between the converter domain/lever arm and the motor domain, and allosterically modulated the dynamics around the ATP binding pocket. Antonovic et al. simulated models of human β-cardiac myosin and fast skeletal myosin IIa in the pre-powerstroke state [23] and found that the OM binding pocket of the skeletal model had a greater volume and solvent accessible surface area than that of the cardiac model. Finally, Auguin et al. complemented their crystallographic study with 720–1020 ns long conventional MD simulations of pre-powerstroke bovine β-cardiac myosin in the apo, OM-bound, and Mava-bound states (all with Mg^2+^.ADP.P_i_ in the active site) [21]. They found that the binding of OM reduced the mobility of the lever arm and the helix-loop-helix motif of the lower 50 kDa domain, as well as the dynamic changes near the phosphate release ‘backdoor’ near switch 1 and switch 2. A comparative analysis with Mava, which binds to the same pocket, showed that the small molecules’ effects on contractility depend on their allosteric effects on the lever arm as well as a delicate combination of the effects on OFF and ON state myosin heads. Thus, computational and experimental studies of OM–myosin interactions indicate that OM acts by allosterically modulating the lever arm positioning as well as the phosphate backdoor. 

Force generation during the powerstroke of myosin results from a tight coupling between ATP hydrolysis and the conformation of myosin’s lever arm [24]. The lever arm position changes dramatically during the power and recovery strokes, but it has also been shown that the lever arm is dynamic within conformational states and that the variance in lever arm orientation varies from one conformational state to the next [25]. The length of the lever arm is related to the filament sliding velocity in the in vitro motility assay [24]. Similarly, the structure and composition of the lever arms and converter domains [26] of the processive myosins involved in cargo transport (e.g., class V, VI, and X myosins), affects their varied orientations relative to the actin filament; step size, and lever arm stiffness [27]. Snoberger et al. performed hybrid conventional/steered MD simulations of wild type (WT) and mutant (R712L) apo near-rigor state myosin. They found that the R712L mutation disrupts the myosin powerstroke by eliminating a salt bridge between the converter and motor domains [28]. In complementary in vitro motility and optical trap experiments, OM rescued the deficient powerstroke in the R712L mutation [28]. Akter et al. modeled the changes to the OM binding site during the post-rigor to pre-powerstroke conformational transition (i.e., the recovery stroke) using steered and umbrella sampling molecular dynamics simulations [29]. They found that the OM binding site undergoes significant remodeling during the transition, and they also identified several low energy intermediate conformers along the recovery stroke. The conformational changes that occur during transitions in the presence of OM have yet to be described.

Available data indicate that the molecules binding to the converter domain/lever arm/motor domain junction operate by modulating the lever arm dynamics in complex ways. However, these dynamics have not been extensively quantified in molecular simulations. Here, we use MD simulations to explore the dynamics of the motor domain/lever arm hinge in the pre-powerstroke state of bovine β-cardiac myosin and quantify the effects of OM on the lever arm dynamics. Our set of simulations includes three apo replicates and three holo replicates (we use apo and holo throughout to refer to the OM-free and OM-bound simulations; all simulations include Mg^2+^.ADP.P_i_ in the active site) and each replicate was 500 ns long (3 μs net sampling). In the pre-powerstroke chemomechanical state (M.ADP.Pi) myosin heads prepare to form the weakly-bound actomyosin complex with the thin filaments. Within the sarcomere, the number of myosin heads populating this state determines the number of heads that are available to participate in the powerstroke. This state is structurally characterized by a primed lever arm orientation, an open actin binding cleft, and Mg^2+^.ADP.P_i_ in the nucleotide binding pocket. This state has been characterized in many myosin isoforms and has been stabilized crystallographically using ADP and Pi analogues (e.g., vanadate), and the X-ray structures of myosin II isoforms from many species share structural characteristics. Prior structural and computational studies of myosin that evaluated the effects of OM binding indicated a strong reduction in the lever arm mobility due to lever arm binding, which we also observe here. We find that OM changes the average lever arm orientation and reduces the conformational variance of the lever arm positioning. Prior studies have also reported variable interactions made between myosin and OM during MD, which we also observe. We report the conformational diversity of myosin–OM interactions, which could aid in the development of additional compounds targeting the OM binding pocket. Finally, we further explore the residue–residue interactions that play roles in the lever arm dynamics of myosin. A primed lever arm orientation is a hallmark of the pre-powerstroke state, and lever arm priming is important for the orientation of myosin heads in the sarcomere as well as for defining the extent of the powerstroke. Prior MD simulations have found that the lever arm can sample multiple diverse conformations in pre-powerstroke myosin S1 + essential light chain (ELC) constructs [21,30]. We use this analysis to understand the allosteric motions involving the converter domain–lever arm, to identify a hotspot of HCM-associated mutations that may disrupt these dynamics, and to consider how lever arm mobility impacts the power and recovery strokes.

## 2. Results

### 2.1. Omecamtiv Mecarbil Stiffened the Lever Arm and Converter Domain

**Figure 1:**
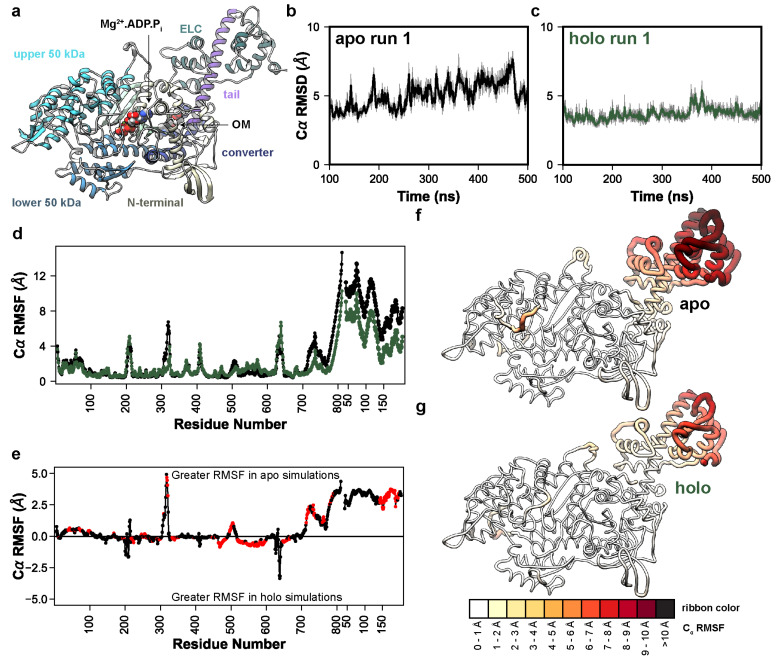
OM allosterically modulates lever arm dynamics in myosin. (**a**) Initial coordinates of OM−bound (holo) myosin used in this study. The major domains are highlighted: N–terminal domain (yellow), upper 50 kDa domain (cyan), lower 50 kDa domain (blue), converter domain (dark blue), tail (purple), central β–sheet (light green), and ELC (green). The time−dependent C_α_ RMSD of the run 1 apo (**b**) and holo (**c**) simulations are shown, highlighting the decrease in conformational fluctuation due to OM binding. (**d**) C_α_ RMSF values are shown as a function of residue number for both myosin and the ELC in the apo (black) and holo (green) simulations. In (**e**) the average difference in RMSF is shown; positive values indicate higher flexibility in the apo simulations and negative values correspond to higher flexibility in the holo simulations. Red points indicate that the difference in RMSF was statistically significant according to a two-tailed Student’s *t*–test (*p* < 0.05). In (**f**,**g**) the per-residue C_α_ RMSF values have been mapped onto the average apo (**f**) and holo (**g**) MD structures. Residues with greater RMSF have thicker ribbons and are colored darker shades of red.

We performed MD simulations of a pre-powerstroke (Mg^2+^.ADP.P_i_-bound) bovine β-cardiac myosin system containing myosin motor domain and tail residues and the ELC (Figure 1a). The motor domain is canonically organized into four subdomains: the N-terminal domain, the upper 50 kDa domain, the lower 50 kDa domain, and the converter domain (highlighted in Figure 1a). To locate the structural regions with conformational variation, we calculated the C_α_ root-mean-square deviation (RMSD) for all C_α_ atoms relative to the OM-bound X-ray structure (PDB: 5N69, Figure 1b,c). The OM-free (apo) and OM-bound (holo) simulations respectively sampled conformations within 5.0 Å (Figure 1b) and 4.1 Å (Figure 1c) of the X-ray structure and there was no statistically significant difference in the RMSD (*p* = 0.08). We also calculated the C_α_ RMSD after aligning to specific C_α_ atoms in the motor domain, converter domain, tail, or ELC (Table 1). Of these subsets, conformational sampling significantly differed between the apo and holo simulations in the converter domain (*p* = 0.05). Next, we used the *mdlovofit* algorithm to identify the 35% C_α_ atoms with the smallest conformational variation in the MD simulations. This was conducted because changes in the structure due to the motor domain/lever arm hinge frustrate the attempts to find an optimal alignment for all atoms. The *mdlovofit* subset includes C_α_ atoms primarily in the motor domain. After identifying this subset, we re-aligned the simulations to the *mdlovofit* subsets and recalculated the C_α_ RMSDs for only the *mdlovofit* subset atoms as well as for all the C_α_ atoms in the simulations. Both calculations indicated statistically significant differences in sampling between the apo and holo simulations (*p* = 0.04 and *p* = 0.01, Table 1). These results indicate that OM binding to pre-powerstroke myosin dampens the conformational fluctuations in the converter domain as well as the relative motions between the tail/ELC and motor domains. After aligning the trajectories to the *mdlovofit* subsets, we also calculated the root-mean-square fluctuations (RMSF) for all C_α_ atoms, which measures the fluctuations of each C_α_ atom relative to its average position (Figure 1d). OM binding decreased the C_α_ RMSFs in the converter domain and tail by ~2 Å in the converter domain. OM also significantly (*p* < 0.05) increased the conformational fluctuations in the helix-loop-helix motif of the lower 50 kDa domain, but the magnitude of this increase was smaller (~0.5 Å) (Figure 1e). Overall, there was a statistically significant difference (*p* < 0.05) in the C_α_ RMSF for 311 residues in our model (Figure 1e). RMSF values were then mapped onto average structures in the simulations: a global analysis of conformational sampling indicates that the tail and ELC had the greatest fluctuations and that these fluctuations were dampened in the presence of OM (Figure 1f,g). Supplemental Movies S1 and S2 highlight changes in the lever arm orientation in the apo and holo simulations, respectively. Supplemental Movies S3 and S4 depict the same dynamics as Movies S1 and S2 but focuses instead on the variation of the motor domain, as the trajectory has been aligned to the C-terminus of the lever arm. 

### 2.2. Omecamtiv Mecarbil Altered Lever Arm Priming

**Figure 2:**
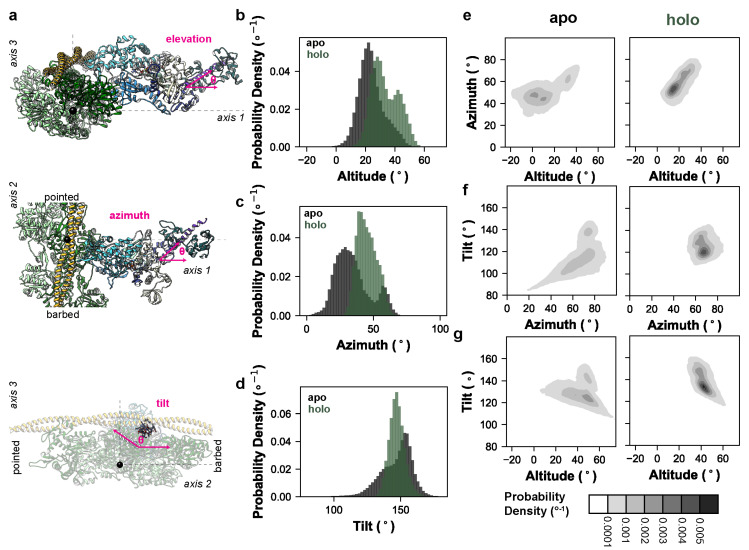
OM impacts myosin’s lever arm orientation and conformational entropy. (**a**) We constructed a coordinate system based on the weakly-bound actomyosin complex. In these models, the myosin ribbon is depicted as in Figure 1a, an actin pentamer is shown in green, and tropomyosin in yellow. One axis corresponds to a line drawn between the center of the thick and thin filaments, the second axis lies parallel to the thick and thin filaments, and the third axis is normal to the plane defined by axes 1 and 2 (dashed lines). Our simulations, which do not explicitly include the thick or thin filaments, were aligned to the lower 50 kDa residues involved in the post-powerstroke actomyosin complex in PDB: 8EFH. The origin is centered at the center of mass of the actin pentamer used to construct this coordinate system (black sphere). We measured three angles (magenta): an elevation angle corresponding to the rise of the lever arm above axis 1, an azimuthal angle corresponding to the position of the lever arm along axis 3, and a tilt angle corresponding to the position of the lever arm relative to axis 3. Probability densities (i.e., integration across all bins = 1, the bins are 2° wide) for the elevation, azimuth, and tilt angles for the apo (black) and holo (green) simulations are shown in panels (**b**–**d**), respectively. Results were aggregated across replicate simulations. The 2D probability densities (2° × 2° bins) for all three combinations of angles for the apo and holo simulations are shown in panels (**e**–**g**).

Given the observed effect of OM on the converter domain/lever arm mobility, we next analyzed the extent to which OM modulated the relative orientation of the motor domain and lever arm. Our simulations sample the conformations in the pre-powerstroke chemomechanical state, which precedes the formation of the weakly-bound actomyosin complex, so we constructed a coordinate system based on the weakly-bound state inspired by the cryo–EM work of Klebl et al. [31]. The first axis of our coordinate system perpendicularly connects the thick and thin filaments (Figure 2a). The second axis is oriented parallel to the thick and thin filaments. The third axis is normal to the plane defined by the first and second axes. The cryo–EM structure in Klebl et al. predicts that the weakly- bound state is characterized by the binding of the lower 50 kDa domain to the thin filament without closure of the actin binding cleft, and that the lower 50 kDa domain–actin interaction is similar to the interaction observed in the post-powerstroke and rigor-like conformations of the actomyosin complex. So, we aligned our simulations on the helix-loop-helix motif of the lower 50 kDa domain to the cryo–EM structure of the post-powerstroke actomyosin complex (PDB ID: 8EFE) [32]. The actin pentamer of the 8EFE structure was centered at the origin and the thin filament was oriented along axis 2. Next, we defined the tail vector based on a line connecting the backbone heavy atoms (C_α_, C, and N) from residues 769–771 to 784–787. Only the N-terminal region of the tail up to the ELC binding site were included because the C-terminal region can curve during the simulations. This crossbridge centric coordinate system should assess the effects of structural perturbations on the orientation of myosin motors in the sarcomere more precisely than those approaches that measure the tail orientation via myosin-centric measures. The angle of the tail was then calculated using three angles: the elevation of the tail relative to axis 1 (35° in the X-ray structure), the azimuth of the tail relative to axis 1 (43° in the X-ray structure), and the tilt of the tail relative to axis 3 (142° in the X-ray structure) (Figure 2a). The holo simulations had a greater average tail elevation relative to the apo simulations (33° vs. 23°, respectively) (Figure 2b). The holo simulations had a greater tail azimuth relative to the apo simulations (45° vs. 34°) (Figure 2c). The holo simulations had approximately equal tilt relative to the apo simulations (147.1° vs. 146.9°) (Figure 2d). For all measured angles, the presence of OM reduced the conformational heterogeneity of the tail (Figure 2b–d). This shift in the tail orientation and reduction in the conformational entropy was further observed in the 2D probability density distributions for all combinations of measured angles (Figure 2e–g). Supplemental Movies S1–S4 depict run 1 of the apo and holo MD trajectories studied here. To better highlight the range of conformations sampled by the lever arm we binned the apo and holo run 1 trajectories according to the value of the elevation angles. The trajectories were binned into twenty 5° wide bins and the coordinates in each bin were averaged. Then, we generated the nonphysical trajectories that morph between the bin-averaged conformers via coordinate interpolation. These movies show the dynamic range of motion for the lever arm along the elevation angle for the apo (Supplemental Movie S5) and holo (Supplemental Movie S6) simulations. For averaging, trajectories were aligned on residues at the motor domain–lever arm junction. Due to the averaging and interpolation procedures, coordinates far from the alignment site become distorted in these movies and reflect the conformational degrees of freedom within each averaging window. 

### 2.3. Conformational Sampling and Interactions Made by Omecamtiv Mecarbil 

**Figure 3:**
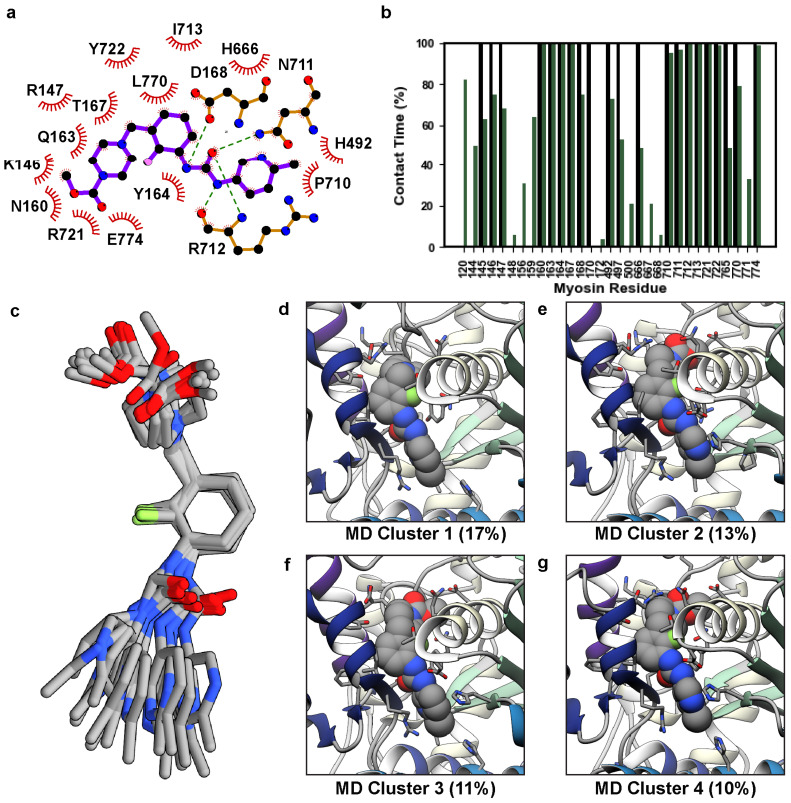
Conformational variations in OM. (**a**) A LigPlot [34] diagram of the 5N69 X-ray structure highlights the interactions created by OM. (**b**) We analyzed the fraction of simulation time (y-axis) for which any OM atom interacted with a myosin residue (x-axis). Black bars always have 100% contact time and correspond to the crystal structure, green bars correspond to the average contact time across three replicate simulations. (**c**) Representative structures for the top 14 MD-derived OM clusters are shown and have been aligned on the central fluorobenzene moiety to highlight the conformational variation in the terminal moieties of OM. Snapshots in panels (**d**–**g**) show the top four MD clusters corresponding to OM’s orientation in its binding pocket.

In the X-ray structure, OM binds to a pocket formed at the junction of the converter domain, lever arm, relay helix, and N-terminal domain of myosin. Both polar and nonpolar interactions participate in the protein–ligand complex that enables OM to structurally connect these regions (Figure 3a). We assessed the structural stability of the protein–ligand interactions in the MD simulations by tracking the percent simulation time for which OM was in contact with any myosin residue. OM was considered in contact with a myosin residue if at least one heavy atom pair were within 5 Å of one another; results were averaged across replicate simulations and presented as an average percent simulation time that a ligand–protein interaction was present. Interactions present for less than 5% simulation time were excluded. Under these criteria, OM formed interactions with 21 residues in the X-ray structure (Figure 3b). In the MD simulations, OM maintained interactions with 20/21 of these residues for at least 15% of any individual simulation and at least 49% of the aggregate simulation time. In the MD simulations OM also formed novel interactions with 10 residues (Figure 3b, Table S1). Of these 10 novel contacts, interactions with residues L120 and E500 were observed in all three simulations; interactions with G144, S156, D159, P667, and H668 were observed in 2/3 simulations; interactions with S148, Q172, and G771 were observed in 1/3 simulations (Table S1). The reproducible and enduring interaction with L120 was made possible by a rotation of the L120 χ_2_ side-chain dihedral angle and allowed hydrophobic interactions with the terminal methyl group of OM. This interaction may anchor OM at the base of its binding pocket. There were two regions where crystallographic contacts were maintained for 100% of the aggregate simulation time. The first corresponds to the interactions between the central moieties of OM (the pyrazine, fluorobenzene, and carbamide) and an α-helix in the N-terminal domain (residues 154–168) that is oriented perpendicular to the lever arm. The second zone includes the first β-strand of the converter (residues 711–714) and R721, which interact with the pyrazine, fluorobenzene, carbamide, and methyl pyridine moieties of OM. The terminal OM moieties (methyl formate and methyl pyridine) created more heterogeneous interactions with the myosin residues and sampled alternate conformations in MD. We used the *NMRclust* algorithm [33] as implemented in *UCSF Chimera* to identify the alternate conformations sampled by OM. These conformers are aligned on the central (i.e., most conformationally homogenous) moieties in Figure 3c. Figure 3a depicts the crystallographic conformation of the pocket and panels 3d-g show the top four most populous conformational clusters observed in MD, which account for ~52% of the conformational variance observed in MD. The methyl formate moiety adopted two distinct conformations: an X-ray like conformer in which it interacted with residues in the lever arm (Figure 3d) and an alternate conformation in which it interacted with the 154–168 α-helix (Figure 3e–g). In the X-ray structure, the methyl pyridine moiety is surrounded by several bulky residues including Y164, H492, H666, and R712. In MD simulations, these interactions were maintained but were fluid: local conformational changes allowed the structural contacts to be maintained in this region, but sampling of alternate conformations was possible. For example, rotation of the H666 side chain allowed rotation of the methyl pyridine (compare Figure 3d,e with Figure 3g,h). 

### 2.4. Residue–Residue Interactions Varied with Lever Arm Orientation 

**Figure 4:**
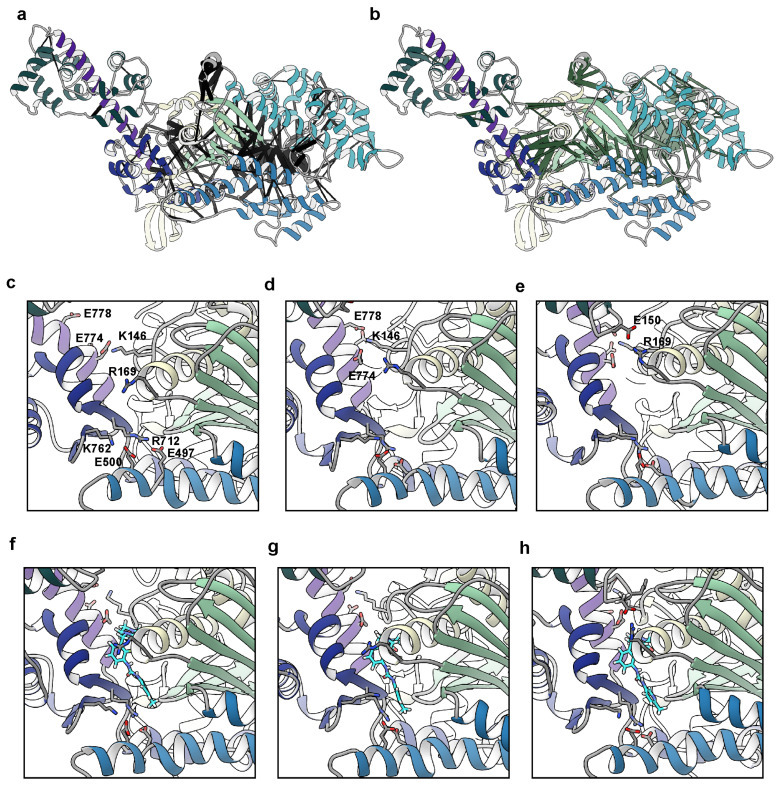
Changes in residue–residue contacts due to OM binding and lever arm motion. We calculated the residue–residue contacts that were present at different probabilities in the apo and holo simulations. Contact pairs that were present for a significantly (*p* < 0.05, two-tailed Student’s *t*-test) greater time in the apo simulations are highlighted with black pseudo-bonds in panel (**a**) and pairs that were dominant in the holo simulations are represented with green pseudo-bonds in panel (**b**). The pseudo-bond radii are proportional to the difference in contact time: thicker pseudo-bonds correspond to greater contact populations. Statistically significant differences in the contact patterns were localized to three regions around the motor domain–lever arm hinge, the transducer, and the phosphate back door. We also explored the residue–residue contacts that varied with the lever arm orientation. Panels c-e focus on the OM binding pocket in the apo simulations at low (**c**), average (**d**), and high (**e**) values of the ‘elevation’ angle. Panels (**f**–**h**) focus on the same region and elevation angles in the holo simulations. Atoms for charged residues with conformation-dependent salt bridges are shown and labeled in panel (**c**). The carbon atoms of OM are cyan in panels (**f**–**h**).

Our simulations indicated that OM primes and stabilizes the lever arm orientation by restricting myosin’s flexibility at the motor domain/lever arm hinge. Additionally, we observed that OM allosterically modulated the flexibility of the helix-loop-helix motif in the lower 50 kDa domain. We next analyzed the residue–residue contacts in our simulations to find the differences associated with OM. There was an average of 9242 residue–residue contact pairs in the apo simulations and 9063 pairs in the holo simulations (*p* = 0.37). We searched for the residue–residue contact pairs that differed significantly (*p* ≤ 0.05) between the apo and holo simulations and for which there was at least a 10% difference in contact time between the apo and holo simulations. This search yielded 719 statistically distinct contacts, 407 of which had at least a 10% difference in contact time. Of these 407 contacts, 213 occurred more frequently in the apo simulations and 194 occurred more frequently in the holo simulations. The application of a 10% difference threshold focuses our analysis on the regions of myosin with major conformational responses to OM binding. We mapped the statistically distinct contacts onto the initial apo structure in Figure 4a,b. Figure S1 provides the maps of contact differences at 5% and 20% difference thresholds for comparison. In the 10% difference threshold map, the different residue–residue pairs localized to three regions: the converter domain/lever arm/N-terminal domain interface, the helix G (residues 217–232)/transducer (residues 245–266)/W helix (residues 647–663) interface, and the switch 1 (residues 231–244)/switch 2 (residues 462–472)/and upper 50 kDa domain loop (residues 275–284) interface. The conformation of the first interface is related to the lever arm orientation while the conformation of the latter two are related to the phosphate back door. We focused on the contacts within the converter domain/lever arm/N-terminal domain region and found that residues in an N-terminal domain loop (residues 142–153) and helix (residues 154–168), the C-terminus of the relay helix (residues 492–503), the N-terminus of the converter (residues 711–723), and the N-terminus of the lever arm (residues 770–781) had interactions that varied with lever arm orientation (Figure 4c–h). This region includes many polar and charged residues and several salt bridges formed/broke as the lever arm rotated. Some interactions, such as the converter domain–relay helix salt bridge between residues K762–E500, were present at more extended lever arm orientations (Figure 4c,f) but were absent in more compact orientations (Figure 4e,h). Other interactions, for example a salt bridge between ELC residue E150 and R169 in the myosin N-terminal domain, were only present in the compact conformations (Figure 4e,h). Some residues (e.g., K143) could form salt bridges with multiple partners depending on the lever arm orientation (e.g., E774, D778). Finally, some interactions, such as the salt bridge between R712–E497, were maintained independently of the lever arm conformation. OM binding reduced the range of motion of the lever arm but also placed conformational constraints on the residue side chain conformations in this region as the lever arm adopted different conformations. 

## 3. Discussion

The development and clinical success of myosin-targeted small molecules to treat myopathies could pave the way for other myotropes to treat a range of cardiac and skeletal myopathies. Intriguingly, similar compounds binding to the same pocket on myosin can have drastically different effects on function and can even target/stabilize different conformational states in the chemomechanical cycle [21]. A greater understanding of conformational sampling within this region (both within specific chemomechanical states and in transitions between them) could aid in the development of novel myotropes or the redesign of existing myotropes for targeted therapeutic purposes. 

Here, we found that the binding of OM to myosin primarily stabilizes the lever arm orientation in the pre-powerstroke conformation, as has been seen in prior X-ray crystallography and molecular dynamics studies [19,20,21]. One effect of the lever arm stabilization was to reorient the motor domain towards the pointed end of the thin filament. Under the assumption that all other structural factors remain the same, our finding suggests that OM binding can prime myosin to have a larger step size due to this reorganization. Another effect was the reduction in the conformational entropy of the tail, which may stabilize the pre-powerstroke state and/or facilitate the formation of the weakly-bound actomyosin complex. However, our model does not include the thick or thin filaments nor are the motor domains constrained by the full, biologically active hetero-hexamer myosin–ELC–RLC complex. These speculative effects of OM on myosin dynamics are better assessed in larger scale, coarser grained models. We also found that myosin allosterically altered the flexibility of the helix-loop-helix region and the contacts near the phosphate release back door, as has been observed in prior MD simulations. These observations across multiple studies, utilizing different starting models and force fields, provide strong evidence for the conformational effects of OM. Here, we explored how such conformational changes impact myosin’s pre-powerstroke orientation. By measuring the lever arm structure within a coordinate system based on a weakly-bound crossbridge, these simulations predict that the binding of OM reorients the primed myosin heads towards the pointed end of the thin filaments. 

The energetic consequences of OM binding have not been explored, and several possible consequences relate to the formation of the weakly-bound crossbridges, the dynamics of the powerstroke, and the stability of the strongly-bound crossbridges. Our simulations suggest that OM may reorient the myosin heads towards binding sites on the thin filaments. Additionally, we identified differences in the helix-loop-helix motif that are thought to form the initial actin–myosin interactions during weak binding. The effects of OM as a ‘glue’ near the converter domain suggests a heightened energy barrier for the powerstroke, but accelerated phosphate release has been observed experimentally in the presence of OM. Pharmacological intervention at the binding pocket between the motor domain and the converter domain/lever arm can exert a powerful influence on crossbridge cycling. The nature of this influence as either activating or inhibiting depends on the specific molecule bound, the extent of the thin filament activation, and the structural organization of the thick filament. The simulations performed here describe the specific conformational changes that occur in the vicinity of the OM binding pocket and the relative orientations of the motor domain and lever arm. However, we can anticipate that small molecules such as OM can modify the myosin structure in other chemomechanical states. The development of more complex structural and dynamic models of the power and recovery strokes in the presence of small molecules such as OM is the next step towards assessing how myosin conformation, as well as other structural factors (e.g., thin filament activation, thick filament organization), can influence crossbridge cycling.

We have characterized the range of motion present in the lever arm orientation in the pre-powerstroke state. While the global free energy minima of the lever arm orientation observed in our simulations was like the crystallographically observed orientations, large departures from this conformation were observed. At a high elevation of the lever arm the ELC formed heterogeneous, transient interactions between the C-terminal lobe of the ELC and the motor domain. At very elevated orientations, the ELC formed interactions with the residues in loop 1, the hairpin turn containing residue G256, and the N-terminal helix containing R169 (Figure 4e,h). This interaction is like the contacts first observed in the pre-powerstroke structures of chicken smooth muscle myosin [35]. Changes in the charge and length of loop 1 have been proposed to modulate the rate of ADP release from myosin [36]. Interaction between the ELC C-terminal lobe and this region may exert some influence on nucleotide handling or the stabilization of the pre-powerstroke conformations that are required for actin binding. Prior studies have also identified potential interactions between the disordered N-terminal extension of the ELC, which was not modeled here, and the SH3 domain of myosin in the ‘down’ positions of the lever arm associated with the post-powerstroke (AM.ADP) state [37,38]. This hinge is a highly dynamic region, due in part to the large druggable pocket that OM can occupy. This pocket is lined with many polar and charged residues. There is a complex interplay between the alternate hydrogen bonding patterns and the protein–solvent interactions. Our simulations were performed in the presence of neutralizing counterions: more complex protein–solvent relationships may manifest at biologically relevant ionic strengths. 

There are many HCM-linked mutations that line the pocket connecting the converter domain, the lever arm, and the motor domain. For example, the R712L mutation is associated with a severe HCM pathology and has been studied [28,39]. We found that the R712–E497 mutation was extremely conserved across all lever arm orientations in our study, and so we can predict that disruption of this salt bridge would have severe effects on the lever arm and the conformational transitions associated with the power/recovery strokes. Other mutations involving charged residues in this region include R143G/W/Q [40,41,42], G144D [43], K146N [44], R147S [45], D168N [43], R169G/K/S [46,47], E497D/G [48], E500A [40], R712L [39], K762R [47], E774V [49], D778N,G,V,E [50,51,52,53], and E779D [54]. Interestingly, all these mutations involving charged residues are associated with HCM phenotypes. Formation of the interacting heads motif and the sequestration of myosin into the super-relaxed state appears to require the heads to adopt a pre-powerstroke orientation with primed lever arms. Disruption of the electrostatic interactions in this region may destabilize the IHM and result in an increased number of heads available to participate in crossbridge cycling. Disruption of the electrostatics in this region may also affect the orientation of the lever arm and/or the energetics of the powerstroke. For now, we can identify that the charged residues in and around the converter domain/lever arm/N-terminal domain junction are an HCM hotspot, and we find that changes in the lever arm structure are related to the distinct hydrogen bonding patterns among the charged residues. Additional studies of this region—particularly the conformational changes and energetics associated with the lever arm—are required. This region is fortuitously druggable, and compounds may variously affect the lever arm orientation (OM and mava) and/or the stability of the IHM (mava only). Understanding the combination of effects that small molecules have on tail allostery and the thermodynamic stabilization of various conformational states in the crossbridge cycle is key to developing more targeted and/or efficacious therapeutics. This represents an opportunity to design variable small molecules to occupy this druggable site and treat several myopathies, depending on which conformational states and transitions are most affected by small molecule binding. 

## 4. Materials and Methods

**Model Preparation:** Structures of pre-powerstroke (M.ADP.P_i_) bovine cardiac myosin were prepared from the 2.45 Å X-ray crystal structure of bovine cardiac β-myosin in complex with the essential light chain, omecamtiv mecarbil (OM), ADP, vanadate, and Mg^2+^ solved by Planelles-Herrero et al. (PDB: 5N69) [20]. Multiple copies of the molecule were present in the unit cell: chains A and H were used. Missing coordinates including terminal residues, loop 1 (residues 201–214), the tip of the cardiomyopathy loop (residues 405–410), loop 2 (residues 623–644), and a loop in the converter (residues 733–735) were constructed using *Modeller* [55]. Multiple loop conformations were obtained using the *loopmodel* routine and the best scoring loops according to the *Modeller* objective function were selected for the final model. We performed a PROCHECK analysis on our model: 91.5% of all residues were in the most favored regions of the Ramachandran space, indicating well-modeled backbone structures, and an overall G-factor of −0.03, indicating ordinary main chain bond geometries. The disordered N-terminal extension of the ELC was not modeled and residues 39–199 are present in our model. The vanadate ion (VO_4_) was replaced by a phosphate ion (H_2_PO_4_). Crystallographic waters and other molecules were removed. Trimethylated lysine residues were converted to lysine. The X-ray study by Planelles-Herrero et al. included a complementary structure of pre-powerstroke myosin in the absence of OM, but residues in the tail and ELC were not resolved (PDB: 5N6A), so we generated an OM-free model by removing the OM ligand from the 5N69 structure and otherwise following the same procedure. His protonation states at pH 7.0 were predicted using the *H++* webserver [56]. A sequence alignment of the human and bovine *myh7* sequences indicated 98% sequence identity for the full sequence and 96% sequence identify for the portion of the structure modeled in our study. None of the variable residues are in direct contact with OM. The human and bovine isoforms also have similar kinetic properties [57]. We have high confidence that the dynamics of the bovine isoform capture OM’s effects on human myosin.

**Molecular Dynamics Simulation:** The resulting systems were prepared for molecular dynamics simulation using the Amber 20 [58] simulation package and the ff14SB force field [59]. Water molecules were treated with the TIP3P force field [60]. Metal ions were modeled using the Li and Merz parameter set [61,62,63]. ADP and Pi (modeled as H_2_PO_4_ [64]) molecules were treated with the GAFF2 force field [65]. Partial charges for ADP and P_i_ were derived from a restrained electrostatic potential (*resp*) fit to quantum mechanics calculations performed with ORCA [66]. Parameters for OM were derived with the AMBER *antechamber* tool. The SHAKE algorithm was used to constrain the motion of hydrogen-containing bonds [67]. Long-range electrostatic interactions were calculated using the particle mesh Ewald (PME) method. Hydrogen atoms were modeled onto the initial structure using the *leap* module of *AMBER* (University of California, San Francisco, California, USA)*,* and each protein was solvated with explicit water molecules in a truncated octahedral box and neutralizing counterions were added. Each system was minimized in three stages. First, hydrogen atoms were minimized for 1000 steps in the presence of 100 kcal/mol restraints on all heavy atoms. Second, all solvent atoms were minimized for 1000 steps in the presence of 25 kcal/mol restraints on all protein atoms. Third, all atoms were minimized for 8000 steps in the presence of 25 kcal/mol restraints on all backbone heavy atoms (N, O, C, and C_α_ atoms). After minimization, systems were heated to 310 K using the NVT (constant number of particles, volume, and temperature) ensemble and in the presence of 25 kcal/mol restraints on backbone heavy atoms. Next, the systems were equilibrated over five successive stages using the NPT (constant number of particles, pressure, and temperature) ensemble. During the first four stages, the systems were equilibrated for 5 ns in the presence of 25 to 1 kcal/mol restraints on backbone heavy atoms. During the final equilibration stage, the systems were equilibrated for 5 ns in the absence of restraints. A 10 Å nonbonded cutoff was used for all preparation and production simulations. The equilibrated systems were then simulated using conventional molecular dynamics protocols in the NVT ensemble in triplicate for 500 ns each (6 total simulations @ 500 ns each = 3 µs total sampling) and coordinates were saved every 10 ps.

**Molecular Analyses:** Because our apo simulations were initiated from a holo-like conformation of the lever arm, we allowed for an extended equilibration period and ignored the first 100 ns of all production simulations. The remaining 400 ns of production dynamics for each simulation were analyzed at 10 ps granularity unless otherwise specified. The C_α_ RMSD, C_α_ RMSF, residue–residue contacts, and lever arm orientations were analyzed with *cpptraj* [68]. Two residues were considered in contact with one another if at least one pair of heavy atoms were within 5 Å of one another. Residue contacts were analyzed at 100 ps granularity. Tail angles were calculated using the vector module of *cpptraj*. C_α_ atoms were chosen for alignment using a Low-Order-Value-Optimization strategy as described by Martínez and coworkers [69,70,71] and implemented in *mdlovofit* (*v20.0.0*) [https://github.com/m3g/MDLovoFit/releases/tag/v20.0.0, accessed on 13 June 2023] [69]. The apo and holo simulations were subsampled every 1 ns, non-protein atoms were removed, and then the two simulations were combined into a single ensemble. We calculated average structures for the aggregate ensemble using *cpptraj*. We used *mdlovofit* to identify the subset of C_a_ atoms with the smallest possible average RMSD to the averaged structures as a function of subset size (F). Protein images and movies were generated with *UCSF Chimera* [72].

## Figures and Tables

**Table 1:** Identification of mobile subdomains via the C_α_ RMSD.

System	Apo (OM-Free)				Holo (OM-Bound)				*p*-Value ^8^
Replica	1	2	3	Avg.	1	2	3	Avg.	
All C_α_^1^	5.1	4.6	5.4	5.0	3.7	4.8	3.7	4.1	0.08
Motor Domain ^2^	3.3	3.1	3.1	3.2	3.2	3.5	3.1	3.3	0.66
Converter ^3^	1.2	1.0	1.1	1.1	2.0	1.7	1.3	1.7	0.05
Tail ^4^	1.8	2.2	2.0	2.0	2.0	1.6	1.6	1.7	0.18
ELC ^5^	2.7	3.0	2.9	2.9	3.0	3.8	2.7	3.1	0.45
MDLOVO subset ^6^	1.0	1.1	1.2	1.1	1.2	1.3	1.3	1.3	0.04
MDLOVO all ^7^	7.3	6.3	7.6	7.1	4.3	5.3	4.5	4.7	0.01

^1^. All C_α_ atoms were used in both the structural alignment and RMSD calculation. ^2^. C_α_ atoms in the motor domain but not the tail and ELC were used for the alignment and RMSD calculation. We considered residue 704 as the end of the motor domain, which excludes the hinge comprised of a flexible turn and the converter domain. ^3^. Converter domain C_α_ atoms in residues 712–766 were used for the alignment and RMSD calculation. To define the converter domain, we selected residues conventionally assigned to this region and excluded terminal residues that adopt a coil or turn secondary structure. ^4^. Tail C_α_ atoms in residues 768–810 were used for the alignment and RMSD calculation. This selection does include terminal residues around the tail that have some flexibility. ^5^. All C_α_ atoms in the ELC were used for the alignment and RMSD calculation. ^6^. The mdlovofit-selected 338 C_α_ atoms were used for the alignment and RMSD calculation. ^7^. The mdlovofit-selected 338 C_α_ atoms were used for the alignment but the RMSD calculation was calculated across all C_α_ atoms. ^8^. *p*-values were calculated with a two-tailed Student’s *t*-test. C_α_ RMSDs were calculated as an average for each replicate simulation and treated as individual observations for each condition.

## Data Availability

MD trajectories presented in this study are housed in the molecular dynamics archives of the University of Washington Center for Translational Muscle Research and are available upon request (https://ctmr.washington.edu, accessed on 26 September 2024).

## Supplementary Materials

The following supporting information can be downloaded at: https://www.mdpi.com/article/10.3390/ijms251910425/s1.

## References

[1] Berg J.S., Powell B.C., Cheney R.E. (2001). A Millennial Myosin Census. Mol. Biol. Cell.

[2] Sellers J.R. (2000). Myosins: A Diverse Superfamily. Biochim. et Biophys. Acta (BBA)-Mol. Cell Res..

[3] Thompson R.F., Langford G.M. (2002). Myosin Superfamily Evolutionary History. Anat. Rec..

[4] Walklate J., Ujfalusi Z., Geeves M.A., Lindstedt S.L., Hoppeler H.H. (2016). Myosin Isoforms and the Mechanochemical Cross-Bridge Cycle. J. Exp. Biol..

[5] Morgan B.P., Muci A., Lu P.-P., Qian X., Tochimoto T., Smith W.W., Garard M., Kraynack E., Collibee S., Suehiro I. (2010). Discovery of Omecamtiv Mecarbil the First, Selective, Small Molecule Activator of Cardiac Myosin. ACS Med. Chem. Lett..

[6] Green E.M., Wakimoto H., Anderson R.L., Evanchik M.J., Gorham J.M., Harrison B.C., Henze M., Kawas R., Oslob J.D., Rodriguez H.M. (2016). A Small-Molecule Inhibitor of Sarcomere Contractility Suppresses Hypertrophic Cardiomyopathy in Mice. Science.

[7] Chuang C., Collibee S., Ashcraft L., Wang W., Wal M.V., Wang X., Hwee D.T., Wu Y., Wang J., Chin E.R. (2021). Discovery of Aficamten (CK-274), a Next-Generation Cardiac Myosin Inhibitor for the Treatment of Hypertrophic Cardiomyopathy. J. Med. Chem..

[8] Voors A.A., Tamby J., Cleland J.G., Koren M., Forgosh L.B., Gupta D., Lund L.H., Camacho A., Karra R., Swart H.P. (2020). Effects of Danicamtiv, a Novel Cardiac Myosin Activator, in Heart Failure with Reduced Ejection Fraction: Experimental Data and Clinical Results from a Phase 2a Trial. Eur. J. Heart Fail..

[9] Olivotto I., Oreziak A., Barriales-Villa R., Abraham T.P., Masri A., Garcia-Pavia P., Saberi S., Lakdawala N.K., Wheeler M.T., Owens A. (2020). Mavacamten for Treatment of Symptomatic Obstructive Hypertrophic Cardiomyopathy (EXPLORER-HCM): A Randomised, Double-Blind, Placebo-Controlled, Phase 3 Trial. Lancet.

[10] Keam S.J. (2022). Mavacamten: First Approval. Drugs.

[11] Maron M.S., Masri A., Nassif M.E., Barriales-Villa R., Arad M., Cardim N., Choudhury L., Claggett B., Coats C.J., Düngen H.-D. (2024). Aficamten for Symptomatic Obstructive Hypertrophic Cardiomyopathy. N. Engl. J. Med..

[12] Kooiker K.B., Mohran S., Turner K.L., Ma W., Martinson A., Flint G., Qi L., Gao C., Zheng Y., McMillen T.S. (2023). Danicamtiv Increases Myosin Recruitment and Alters Cross-Bridge Cycling in Cardiac Muscle. Circ. Res..

[13] Ráduly A.P., Sárkány F., Kovács M.B., Bernát B., Juhász B., Szilvássy Z., Porszász R., Horváth B., Szentandrássy N., Nánási P. (2022). The Novel Cardiac Myosin Activator Danicamtiv Improves Cardiac Systolic Function at the Expense of Diastolic Dysfunction In Vitro and In Vivo: Implications for Clinical Applications. Int. J. Mol. Sci..

[14] Teerlink J.R., Diaz R., Felker G.M., McMurray J.J.V., Metra M., Solomon S.D., Adams K.F., Anand I., Arias-Mendoza A., Biering-Sørensen T. (2020). Cardiac Myosin Activation with Omecamtiv Mecarbil in Systolic Heart Failure. N. Engl. J. Med..

[15] Felker G.M., Solomon S.D., Claggett B., Diaz R., McMurray J.J.V., Metra M., Anand I., Crespo-Leiro M.G., Dahlström U., Goncalvesova E. (2022). Assessment of Omecamtiv Mecarbil for the Treatment of Patients With Severe Heart Failure. JAMA Cardiol..

[16] Kampourakis T., Zhang X., Sun Y., Irving M. (2018). Omecamtiv Mercabil and Blebbistatin Modulate Cardiac Contractility by Perturbing the Regulatory State of the Myosin Filament. J. Physiol..

[17] Malik F.I., Hartman J.J., Elias K.A., Morgan B.P., Rodriguez H., Brejc K., Anderson R.L., Sueoka S.H., Lee K.H., Finer J.T. (2011). Cardiac Myosin Activation: A Potential Therapeutic Approach for Systolic Heart Failure. Science.

[18] Liu Y., White H.D., Belknap B., Winkelmann D.A., Forgacs E. (2015). Omecamtiv Mecarbil Modulates the Kinetic and Motile Properties of Porcine β-Cardiac Myosin. Biochemistry.

[19] Winkelmann D.A., Forgacs E., Miller M.T., Stock A.M. (2015). Structural Basis for Drug-Induced Allosteric Changes to Human β-Cardiac Myosin Motor Activity. Nat. Commun..

[20] Planelles-Herrero V.J., Hartman J.J., Robert-Paganin J., Malik F.I., Houdusse A. (2017). Mechanistic and Structural Basis for Activation of Cardiac Myosin Force Production by Omecamtiv Mecarbil. Nat. Commun..

[21] Auguin D., Robert-Paganin J., Réty S., Kikuti C., David A., Theumer G., Schmidt A.W., Knölker H.-J., Houdusse A. (2024). Omecamtiv Mecarbil and Mavacamten Target the Same Myosin Pocket despite Opposite Effects in Heart Contraction. Nat. Commun..

[22] Hashem S., Tiberti M., Fornili A. (2017). Allosteric Modulation of Cardiac Myosin Dynamics by Omecamtiv Mecarbil. PLoS Comput. Biol..

[23] Antonovic A.K., Ochala J., Fornili A. (2023). Comparative Study of Binding Pocket Structure and Dynamics in Cardiac and Skeletal Myosin. Biophys. J..

[24] Uyeda T.Q., Abramson P.D., Spudich J.A. (1996). The Neck Region of the Myosin Motor Domain Acts as a Lever Arm to Generate Movement. Proc. Natl. Acad. Sci. USA.

[25] Trivedi D.V., Muretta J.M., Swenson A.M., Davis J.P., Thomas D.D., Yengo C.M. (2015). Direct Measurements of the Coordination of Lever Arm Swing and the Catalytic Cycle in Myosin V. Proc. Natl. Acad. Sci. USA.

[26] Ménétrey J., Llinas P., Mukherjea M., Sweeney H.L., Houdusse A. (2007). The Structural Basis for the Large Powerstroke of Myosin VI. Cell.

[27] Sun Y., Goldman Y.E. (2011). Lever-Arm Mechanics of Processive Myosins. Biophys. J..

[28] Snoberger A., Barua B., Atherton J.L., Shuman H., Forgacs E., Goldman Y.E., Winkelmann D.A., Ostap E.M. (2021). Myosin with Hypertrophic Cardiac Mutation R712L Has a Decreased Working Stroke Which Is Rescued by Omecamtiv Mecarbil. eLife.

[29] Akter F., Ochala J., Fornili A. (2023). Binding Pocket Dynamics along the Recovery Stroke of Human β-Cardiac Myosin. PLoS Comput. Biol..

[30] Nandwani N., Bhowmik D., Childers M.C., Goluguri R.R., Dawood A., Regnier M., Spudich J.A., Ruppel K.M. (2024). Hypertrophic Cardiomyopathy Mutations Y115H and E497D Disrupt the Folded-Back State of Human β-Cardiac Myosin Allosterically. bioRxiv.

[31] Klebl D.P., McMillan S.N., Risi C., Forgacs E., Virok B., Atherton J.L., Stofella M., Winkelmann D.A., Sobott F., Galkin V.E. (2024). Swinging Lever Mechanism of Myosin Directly Demonstrated by Time-Resolved CryoEM. bioRxiv.

[32] Doran M.H., Rynkiewicz M.J., Rassici D., Bodt S.M.L., Barry M.E., Bullitt E., Yengo C.M., Moore J.R., Lehman W. (2023). Conformational Changes Linked to ADP Release from Human Cardiac Myosin Bound to Actin-Tropomyosin. J. Gen. Physiol..

[33] Kelley L.A., Gardner S.P., Sutcliffe M.J. (1996). An Automated Approach for Clustering an Ensemble of NMR-Derived Protein Structures into Conformationally Related Subfamilies. Protein Eng. Des. Sel..

[34] Laskowski R.A., Swindells M.B. (2011). LigPlot+: Multiple Ligand–Protein Interaction Diagrams for Drug Discovery. J. Chem. Inf. Model..

[35] Dominguez R., Freyzon Y., Trybus K.M., Cohen C. (1998). Crystal Structure of a Vertebrate Smooth Muscle Myosin Motor Domain and Its Complex with the Essential Light Chain Visualization of the Pre–Power Stroke State. Cell.

[36] Spudich J.A. (1994). How Molecular Motors Work. Nature.

[37] Lowey S., Saraswat L.D., Liu H., Volkmann N., Hanein D. (2007). Evidence for an Interaction between the SH3 Domain and the N-Terminal Extension of the Essential Light Chain in Class II Myosins. J. Mol. Biol..

[38] Landim-Vieira M., Childers M.C., Wacker A.L., Garcia M.R., He H., Singh R., Brundage E.A., Johnston J.R., Whitson B.A., Chase P.B. (2022). Post-Translational Modification Patterns on β-Myosin Heavy Chain Are Altered in Ischemic and Nonischemic Human Hearts. eLife.

[39] Sakthivel S., Joseph P.K., Tharakan J.M., Vosberg H., Rajamanickam C. (2000). A Novel Missense Mutation (R712L) Adjacent to the “Active Thiol” Region of the Cardiac Β-myosin Heavy Chain Gene Causing Hypertrophic Cardiomyopathy in an Indian Family. Hum. Mutat..

[40] Mohiddin S.A., Begley D.A., McLam E., Cardoso J.-P., Winkler J.B., Sellers J.R., Fananapazir L. (2003). Utility of Genetic Screening in Hypertrophic Cardiomyopathy: Prevalence and Significance of Novel and Double (Homozygous and Heterozygous) -Myosin Mutations. Genet. Test..

[41] Erdmann J., Daehmlow S., Wischke S., Senyuva M., Werner U., Raible J., Tanis N., Dyachenko S., Hummel M., Hetzer R. (2003). Mutation Spectrum in a Large Cohort of Unrelated Consecutive Patients with Hypertrophic Cardiomyopathy. Clin. Genet..

[42] Kimura A., Ito-Satoh M., Hayashi T., Takahashi M., Arimura T. (2001). Molecular Etiology of Idiopathic Cardiomyopathy in Asian Populations. J. Cardiol..

[43] Homburger J.R., Green E.M., Caleshu C., Sunitha M.S., Taylor R.E., Ruppel K.M., Metpally R.P.R., Colan S.D., Michels M., Day S.M. (2016). Multidimensional Structure-Function Relationships in Human β-Cardiac Myosin from Population-Scale Genetic Variation. Proc. Natl. Acad. Sci. USA.

[44] Ingles J., Doolan A., Chiu C., Seidman J., Seidman C., Semsarian C. (2005). Compound and Double Mutations in Patients with Hypertrophic Cardiomyopathy: Implications for Genetic Testing and Counselling. J. Med. Genet..

[45] Chiou K.-R., Chu C.-T., Charng M.-J. (2015). Detection of Mutations in Symptomatic Patients with Hypertrophic Cardiomyopathy in Taiwan. J. Cardiol..

[46] Pan S., Caleshu C.A., Dunn K.E., Foti M.J., Moran M.K., Soyinka O., Ashley E.A. (2018). Cardiac Structural and Sarcomere Genes Associated With Cardiomyopathy Exhibit Marked Intolerance of Genetic Variation. Circ. Cardiovasc. Genet..

[47] Alfares A.A., Kelly M.A., McDermott G., Funke B.H., Lebo M.S., Baxter S.B., Shen J., McLaughlin H.M., Clark E.H., Babb L.J. (2015). Results of Clinical Genetic Testing of 2,912 Probands with Hypertrophic Cardiomyopathy: Expanded Panels Offer Limited Additional Sensitivity. Genet. Med..

[48] Arad M., Penas-Lado M., Monserrat L., Maron B.J., Sherrid M., Ho C.Y., Barr S., Karim A., Olson T.M., Kamisago M. (2005). Gene Mutations in Apical Hypertrophic Cardiomyopathy. Circulation.

[49] Moric E., Mazurek U., Połońska J., Domal-Kwiatkowska D., Smolik S., Kozakiewicz K., Tendera M., Wilczok T. (2003). Three Novel Mutations in Exon 21 Encoding Beta-Cardiac Myosin Heavy Chain. J. Appl. Genet..

[50] Driest S.L.V., Jaeger M.A., Ommen S.R., Will M.L., Gersh B.J., Tajik A.J., Ackerman M.J. (2004). Comprehensive Analysis of the Beta-Myosin Heavy Chain Gene in 389 Unrelated Patients With Hypertrophic Cardiomyopathy. J. Am. Coll. Cardiol..

[51] Lu C., Wu W., Liu F., Yang K., Li J., Liu Y., Wang R., Si N., Gao P., Liu Y. (2018). Molecular Analysis of Inherited Cardiomyopathy Using next Generation Semiconductor Sequencing Technologies. J. Transl. Med..

[52] Otsuka H., Arimura T., Abe T., Kawai H., Aizawa Y., Kubo T., Kitaoka H., Nakamura H., Nakamura K., Okamoto H. (2012). Prevalence and Distribution of Sarcomeric Gene Mutations in Japanese Patients With Familial Hypertrophic Cardiomyopathy. Circ. J..

[53] Harada H., Kimura A., Nishi H., Sasazuki T., Toshima H. (1993). A Missense Mutation of Cardiac β-Myosin Heavy Chain Gene Linked to Familial Hypertrophic Cardiomyopathy in Affected Japanese Families. Biochem. Biophys. Res. Commun..

[54] Millat G., Bouvagnet P., Chevalier P., Dauphin C., Jouk P.S., Costa A.D., Prieur F., Bresson J.-L., Faivre L., Eicher J.-C. (2010). Prevalence and Spectrum of Mutations in a Cohort of 192 Unrelated Patients with Hypertrophic Cardiomyopathy. Eur. J. Med. Genet..

[55] Webb B., Sali A. (2016). Comparative Protein Structure Modeling Using MODELLER. Curr. Protoc. Protein Sci..

[56] Anandakrishnan R., Aguilar B., Onufriev A.V. (2012). H++ 3.0: Automating PK Prediction and the Preparation of Biomolecular Structures for Atomistic Molecular Modeling and Simulations. Nucleic Acids Res..

[57] Deacon J.C., Bloemink M.J., Rezavandi H., Geeves M.A., Leinwand L.A. (2012). Identification of Functional Differences between Recombinant Human α and β Cardiac Myosin Motors. Cell. Mol. Life Sci..

[58] Case D.A., Belfon K., Ben-Shalom I.Y., Brozell S.R., Cerutti D.S., Cheatham T.E., Cruzeiro V.W.D., Darden T.A., Duke R.E., Giambasu G. (2020). AMBER 2020.

[59] Maier J.A., Martinez C., Kasavajhala K., Wickstrom L., Hauser K.E., Simmerling C. (2015). Ff14SB: Improving the Accuracy of Protein Side Chain and Backbone Parameters from Ff99SB. J. Chem. Theory Comput..

[60] Jorgensen W.L., Chandrasekhar J., Madura J.D., Impey R.W., Klein M.L. (1983). Comparison of Simple Potential Functions for Simulating Liquid Water. J. Chem. Phys..

[61] Li P., Song L.F., Merz K.M. (2014). Parameterization of Highly Charged Metal Ions Using the 12-6-4 LJ-Type Nonbonded Model in Explicit Water. J. Phys. Chem. B.

[62] Li P., Song L.F., Merz K.M. (2015). Systematic Parameterization of Monovalent Ions Employing the Nonbonded Model. J. Chem. Theory Comput..

[63] Li P., Merz K.M. (2013). Taking into Account the Ion-Induced Dipole Interaction in the Nonbonded Model of Ions. J. Chem. Theory Comput..

[64] Kiani F.A., Fischer S. (2014). Catalytic Strategy Used by the Myosin Motor to Hydrolyze ATP. Proc. Natl. Acad. Sci. USA.

[65] He X., Man V.H., Yang W., Lee T.-S., Wang J. (2020). A Fast and High-Quality Charge Model for the next Generation General AMBER Force Field. J. Chem. Phys..

[66] Neese F., Wennmohs F., Becker U., Riplinger C. (2020). The ORCA Quantum Chemistry Program Package. J. Chem. Phys..

[67] Miyamoto S., Kollman P.A. (1992). Settle: An Analytical Version of the SHAKE and RATTLE Algorithm for Rigid Water Models. J. Comput. Chem..

[68] Roe D.R., Cheatham T.E. (2013). PTRAJ and CPPTRAJ: Software for Processing and Analysis of Molecular Dynamics Trajectory Data. J. Chem. Theory Comput..

[69] Martínez L. (2015). Automatic Identification of Mobile and Rigid Substructures in Molecular Dynamics Simulations and Fractional Structural Fluctuation Analysis. PLoS ONE.

[70] Martínez L., Andreani R., Martínez J.M. (2007). Convergent Algorithms for Protein Structural Alignment. BMC Bioinform..

[71] Andreani R., Martínez J.M., Martínez L., Yano F. (2008). Continuous Optimization Methods for Structure Alignments. Math. Program..

[72] Pettersen E.F., Goddard T.D., Huang C.C., Couch G.S., Greenblatt D.M., Meng E.C., Ferrin T.E. (2004). UCSF Chimera?A Visualization System for Exploratory Research and Analysis. J. Comput. Chem..

