# Supplementary material for: Dynamics of the Pre-Powerstroke Myosin Lever Arm and the Effects of Omecamtiv Mecarbil
Source: Int J Mol Sci. 2024 Sep 27;25(19):10425. doi: 10.3390/ijms251910425 (PMC11477208; doi:10.3390/ijms251910425)
Supplement: Supplementary file 1 [file ijms-25-10425-s001.zip › ijms-3222112-supplementary.pdf]

Supplemental Material: Dynamics of the pre-powerstroke myosin lever arm and effects of omecamtiv mecarbil

Table S1. Frequency of omecamtiv mecarbil – myosin residue interactions in crystallographic and simulated ensembles

	OM – Myosin Contact Frequency (% Ensemble [1])					
	X-ray	Molecular Dynamics				
Myosin Residue	Chain A	Run 1	Run 2	Run 3	Average	St. Dev.
L 120	0	99	98	48	82	29
G 144	0	51	0	98	50	49
K 145	100	48	41	100	63	32
K 146	100	35	91	98	75	35
R 147	100	96	88	19	68	42
S 148	0	0	19	0	6	11
S 156	0	0	64	30	31	32
D 159	0	0	93	100	64	56
N 160	100	100	100	100	100	0
Q 163	100	100	100	100	100	0
Y 164	100	100	100	100	100	0
T 167	100	100	100	100	100	0
D 168	100	56	69	99	75	22
E 170	100	0	0	0	0	0
Q 172	0	0	0	12	4	7
H 492	100	74	70	76	73	3
E 497	100	75	53	30	53	23
E 500	0	21	31	12	21	10
H 666	100	15	35	96	49	42
P 667	0	0	13	49	21	25
H 668	0	0	6	12	6	6
P 710	100	100	100	86	95	8
N 711	100	100	100	91	97	5
R 712	100	100	100	100	100	0
I 713	100	100	100	100	100	0
R 721	100	99	100	100	100	1
Y 722	100	100	99	99	99	1
F 765	100	49	47	52	49	3
L 770	100	100	36	100	79	37
G 771	0	99	0	0	33	57
E 774	100	99	99	100	99	1

1. OM-myosin interaction times are expressed as the % of a structural ensemble for which a contact was present. A single structure was used for the X-ray structure so values are 0 or 100. The MD ensemble values reflect the percentage of frames for which a contact was present

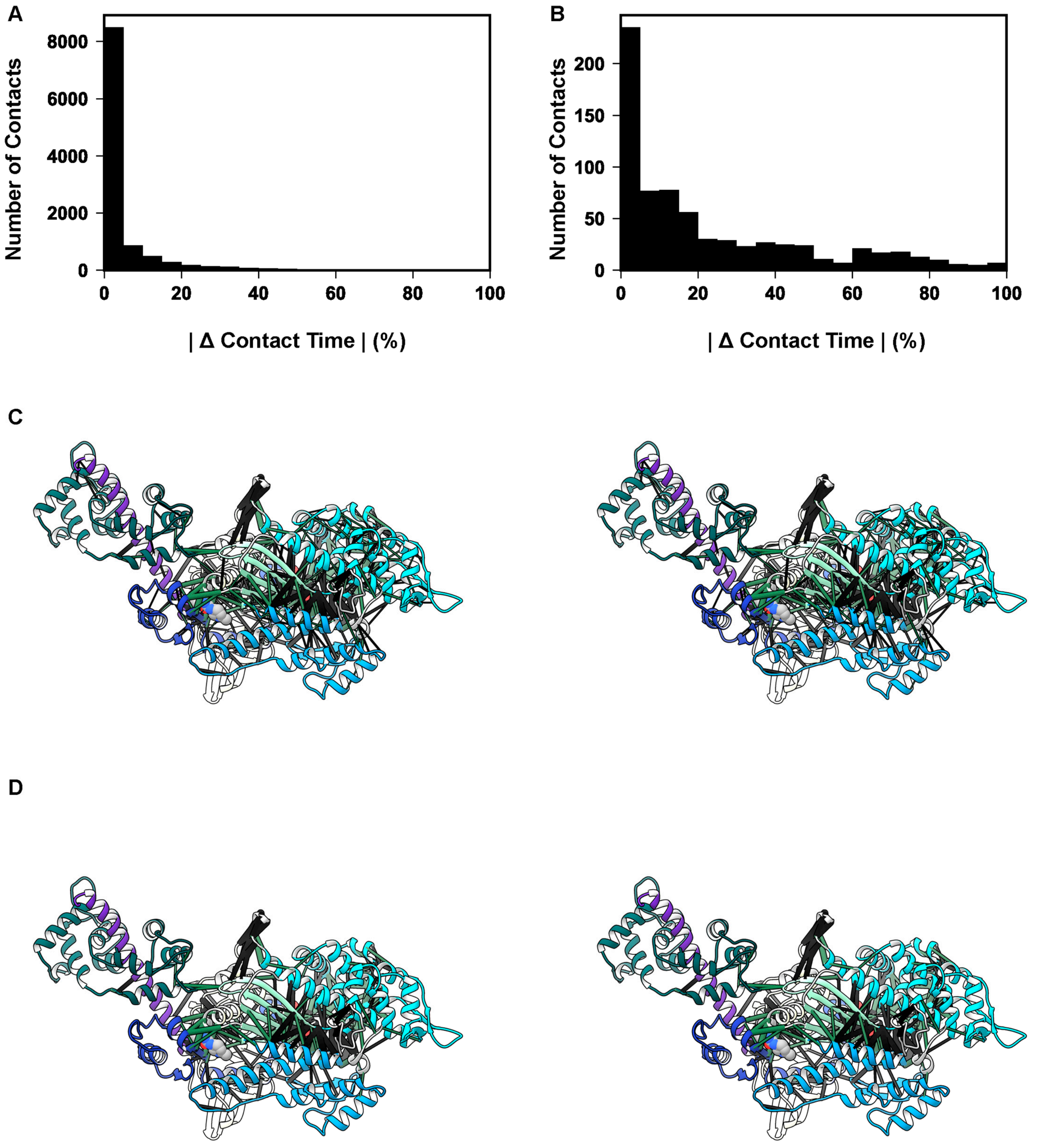

Figure S1. Contact changes in the apo and holo simulations visualized at 5% and 20% difference thresholds. We calculated the absolute value of the difference in % contact time between the apo and holo simulation and then created histograms of the contact difference distribution for all residue-residue contacts (A) and statistically distinct ($p <= 0.05$) residue-residue contacts (B). The elbow point for these distributions is in the 5%-15% range. Contact difference analysis using a 10% difference threshold identified several regions with altered residue-residue interactions due to OM binding. The protein structures in (C) and (D) map differences in

contacts between the apo (black) and holo (green) simulations using 5% and 20% difference thresholds, respectively. The color of the pipes indicates whether the contact was observed more frequently in the apo or holo simulations and pipe radii are proportional to the difference in contact time (larger pipes correspond to larger differences in contact frequency). The images in (C) and (D) contain pipes for both the apo and holo simulations and are presented as cross-eye stereo images.
